# News

**DOI:** 10.1155/S1463924678000164

**Published:** 1978


### News

### Automation and Atomic Spectroscopy

There are few societies whose roots lie in automation. One of them, the Automatic Methods Group of the Chemical Society, London Analytical Division, is holding its annual general meeting on December 7th 1978 in conjunction with the Atomic Spectroscopy Group. Afterwards there will be a meeting entitled *Automation and Atomic Spectroscopy.* This will be at 2 p.m. in the Geological Society Lecture Theatre, Burlington House, London W1. The papers will include: The determination of trace metals in foods using an automated digestion and extraction system followed by plasma emission spectrometry by Dr J. Warren; Computer assisted geochemical analysis with a Pye Unicam SP900 by Dr Baxter; and the automatic analysis of solid samples by laser evaporation coupled to plasma-emission spectroscopy by Dr M. D. Silvester.

Further information may be obtained from D. G. Porter, Laboratory of the Government Chemist, Cornwall House, Stamford Street, London, SE1 9NQ, UK.

### Automation at SAC 80

The Chemical Society, London, Analytical Division have distributed the first circular giving information about SAC 80, their international conference to be held at the University of Lancaster, July 20-26, 1980. Included in the scientific programme will be a session on automation, including the use of microprocessors and mini-computers.

Further information is available from The Secretary, Analytical Division, The Chemical Society, Burlington House, London W1V 0BN, UK.

### Solutions to information handling

A Seminar on *In-house computer solutions to information handling* is to be held on October 31 and November 1, 1978 at the National Computing Centre in Manchester, UK. The programme will include practical aspects of on-line computing and will answer the following questions: have you got an internal information handling problem? Do you need to know more about terminals and other equipment used in on-line searching? Are you familiar with on-line jargon? How do you adapt in-house data bases for on-line searching? What are minicomputers? How can they be used in on-line systems? The ability to retrieve complete papers on-line, instead of abstracts seems an attractive proposition, do microforms provide an answer? What of the future? Will microprocessors be the in-house on-line computers of the future and what part will word processors play in information handling? If you would like full details contact the Seminar Secretary, Mr G. Cousins, Technical Information Unit, ICI Pharmaceuticals Division, Mereside, Alderley Park, Macclesfield, Cheshire, SK10 4TG, UK.

### Selecting small motors for control applications

The Electrical Research Association is organising one of its regular technology transfer seminars to review the basic characteristics of small control motors. The seminar is a one-day event and will be held at the Royal Garden Hotel, London, UK, on Wednesday, 31st January 1979.

The continuing development of small control motors has resulted in situations where equipment designers frequently have a choice between two or more motors with widely different principles of operation and yet similar performance characteristics. The basic characteristics of four main types of small motor for control applications, namely, permanent magnet, d.c. torque, stepping, and d.c. pancake motors, will be considered and the special drive circuits required by some of these motors will be examined. The main theme will be an investigation of the real advantages and limitations in the use of differing motors, and discussion of some typical and unusual applications. Consideration of the interaction of the motor with the drive system and the mechanical load will be included.

The programme for the seminar will be of interest to all concerned with the design, procurement and installation of control systems incorporating small motors, and will cover equipment design, hardware specification, environmental limitations, and future developments. Further information may be obtained from Michael Webb or Muriel Hunns, Seminar Organisers, Electrical Research Association Limited, Cleeve Road, Leatherhead, Surrey KT22 7SA, UK.

### Design of microprocessor-based systems

A series of courses, aimed at designers and engineers entitled, *The Design of Microprocessor-based Systems* has been booked to take place at four different locations throughout England during November 1978. Each complete course will last three days; each day's sessions comprising a self-contained package, able to stand alone, yet complementary to the other sessions. Participants can attend one, two or three days although, of course, the complete three-day syllabus offers the most comprehensive grounding on the topic. Booking forms, a detailed course programme and further information is available from the Organisers; Prodex (Seminars) Ltd., 79 High Street, Tunbridge Wells, Kent TN1 1XZ, UK.

### MicroTest '79

Arrangements for MicroTest '79 are now under way and a first announcement and call for papers will be circulated to potential participants. MicroTest '79 will deal with the testing, maintenance and reliability of equipment using microprocessors and similar LSI circuitry.

Organised by the Society of Electronic and Radio Technicians, in association with the Microprocessor Application Group of the Institution of Electrical Engineers and The Institution of Electrical and Radio Engineers, this residential symposium will be held from 2nd to 4th April 1979 at the University of Sussex, Brighton, UK. For further information contact C. R. Holland, Society of Electronic and Radio Technicians, Faraday House, 8-10 Charing Cross Road, London WC2H 0HP, UK.

### Communications system solves medical centre's anticipated growth needs

A minicomputer serves as a communications concentrator for the Bowman Gray School of Medicine at the North Carolina Baptist Medical Centre. The Data General NOVA 3 allows terminals to be added at various clinics in the hospital without increasing terminal response time. In addition to verifying insurance information in a time-sharing environment, the computer system prints out claim forms in a batch sequence, and provides complete patient histories from previous admissions. It also performs accounting, general ledger, accounts payable, and accounts receivable functions.

Baptist Hospital/Bowman Gray is a regional medical centre with 650 beds and 23,123 patient admissions a year. An additional 206,547 ambulatory patient visits were made to the centre last year. The medical centre is involved in the education of 379 students pursuing the MD, MS, or PhD degrees as well as students studying in several allied health programmes.

Anticipated future growth at the medical centre created the need for a computer system which could adapt to increasing demands on its capabilities. The computer system consists of a Data General NOVA 3 minicomputer configured as a communications concentrator for a Honeywell 6000 host computer. The system presently provides 55 terminals with a two-to-five-second response time. The medical centre can expand the system with the simple addition of terminals, cabling and minor additions to the NOVA, without increasing the terminal response time.

Communications between the NOVA 3 and the Honeywell 6000 host is accomplished synchronously on two 9.6 K-baud lines. The NOVA 3 can communicate asynchronously with up to 75 DASHER display terminals and an additional 80 printers.

Approximately half of the terminals are located in doctors' clinics in the medical centre, where they are used for professional billing, retrieving pertinent patient information, and verifying insurance claim information. Since each clinic operates as an individual entity as part of the Bowman Gray School of Medicine, the computer system must handle 26 different clinics staffed by a total of 130 doctors, each of whom can bill patients.

Future plans at the medical centre are to add on-line admissions, medical record retrieval, dietary restrictions, laboratory tests, and other departments to the system, and to use some of the resulting data for statistical surveys.

### Automatic methods of analysis

A summer school on Automatic Methods of Analysis is being organised by the Chemical Society, UK at Swansea, Wales, July 9-13 1979. Automation is receiving increasing interest in analytical chemistry. This summer school is designed to acquaint laboratory managers, technical staff etc. with both the recent developments in the field as well as with those important aspects of background philosophy and the economic rationale. In addition to lectures, the residential course will provide laboratory sessions in which the student can use a range of the most up to date equipment. There will also be tutorial sessions and a group problem will be set, and students will carry out a systems study on a real and complicated analytical problem. Experience of the laboratory, who have calculated the problem will be made available to participants so that they can discuss in depth the various strategies. Lectures and tutorials will be given by international experts in automation.

Further information is available from D. G. Porter, Laboratory of the Government Chemist, Cornwall House, Stamford Street, London SE1 9NG, UK.

### Calendar

### 1978

**Automation in Microbiology**
November 20-21, Chicago, USA.
*Robert S. First Inc., 405 Lexington Avenue, New York, NY 10017, USA.*

**Automation and Atomic Spectroscopy**
December 7, London, UK.
*D. Porter, Laboratory of the Government Chemist, Cornwall House, Stamford Street, London SE1 9NQ, UK.*

**8th Technicon International Congress: Laboratory Management and Automation**
December 12-14, London, UK.
*Technicon Instruments Co. Ltd., Evans House, Hamilton Close, Houndmills, Basingstoke, RG21 1BZ, Hampshire, UK.*

### 1979

**6th Annual Reports on Analytical Atomic Spectroscopy Symposium**
January 4, Sheffield, UK.
*Miss P. E. Hutchinson, The Chemical Society, Burlington House, London W1V 0BN, UK.*

**Optical Data Display Processing & Storage**
January 23-26, Orlando, Florida, USA.
*Society of Photographic Scientists and Engineers, 1411 K Street, NW, Suite 930, Washington, D.C. 20005, USA.*

**Microsystems '79: an overview of microprocessor technology and applications**
January 31-February 2, London, UK.
Iliffe Promotions Ltd., Dorset House, Stamford Street, London, SE1 9LU, UK.

**The Applications of Microprocessors: A review lecture by Professor J. H. Westcott**
February 8, London, UK.
*The Executive Secretary, The Royal Society, 6 Carlton House Terrace, London SW1Y 5AG, UK.*

**Use of the Microprocessor as a Component in Measurement and Control Equipment**
February 13-15, Stevenage, Herts., UK.
*Carole Meads, Sira Institute, South Hill, Chislehurst, Kent, UK.*

**30th Annual Conference on Analytical Chemistry and Applied Spectroscopy**
5-9 March, Cleveland, USA.
*Mrs. L. Briggs, 437 Donald Road, Pittsburgh, Passadena 15235, USA.*

**International Conferences on Spectroscopy**
29-30 March, Miami, USA.
*Vijay Mohan Bhatnagar, Alena Enterprises of Canada, P.O. Box 1779, Cornwall, Ontario, Canada K6H 5V7.*

**Kongress der Deutschen Gesellschaft fur Laboratoriumsmedizin**
March 29-April 3, Berlin, Germany.
*Wilhelm Syborg, Kongresshall Berlin, John-Foster-Dulles-Allee 10, 1000 Berlin 21, West Germany.*

**3rd International Symposium on Capillary Chromatography**
April 30-May 3, Hindelang/Allgau, W. Germany
*R. E. Kaiser, P.O. Box 1308, D-6702 Bad Durkheim 1, Germany.*

**3rd European Congress of Clinical Chemistry**
June 4-8, Brighton, UK.
*Dr P. J. N. Howarth, Department of Chemical Pathology, Guy's Hospital, Medical School, London, SE1 9RT, UK.*

**2nd World Chromatography Conference**
June 5-6, Lisbon, Portugal.
*V. M. Bhatnagar, P.O. Box 1779, Cornwall, Ontario K6H 5V7, Canada.*

**10th International Symposium on Chromatography and Electrophoresis**
June 19-20, Venice, Italy.
*Dr Alberto Frigerio, Instituto di Riccerche Farmacologiche "Mario Negri", Via Eritrea 62, 20157 Milan.*

**8th International Conference on Atomic Spectroscopy**
July 1-6, Cambridge
*Association of British Spectroscopists, P.O. Box 109, Cambridge, CB1 2HY, UK.*

**2nd Canadian World Chromatography Conference**
July 5-6, Toronto, Canada.
*V. M. Bhatnagar, P.O. Box 1779, Cornwall, Ontario K6H 5V7, Canada.*

**Summer School on Automatic Methods of Analysis**
July 9-13, Swansea, UK.
*D. Porter, Laboratory of the Government Chemist, Cornwall House, Stamford Street, London SE1 9NQ, UK.*

**Analysis '79: Automation in Industrial and Clinical Chemistry.**
July 16-18, London, UK.
*Scientific Symposia Ltd., 33-35 Bowling Green Lane, London, EC1R 0DA, UK.*

